# Coping strategies in patients with good outcome but chronic fatigue after aneurysmal subarachnoid hemorrhage

**DOI:** 10.1007/s00701-023-05549-y

**Published:** 2023-03-13

**Authors:** Hajar Ghafaji, Tonje Haug Nordenmark, Elin Western, Wilhelm Sorteberg, Tanja Karic, Angelika Sorteberg

**Affiliations:** 1 Department of Neurosurgery, Oslo University Hospital, Oslo, Norway; 2 Faculty of MedicineInstitute of Clinical Medicine, University of Oslo, Oslo, Norway; 3 Department of Physical Medicine and Rehabilitation, Oslo University Hospital, Oslo, Norway; 4 Department of Psychology, University of Oslo, Oslo, Norway

**Keywords:** Aneurysmal subarachnoid haemorrhage, Fatigue, Coping, Depression, Anxiety, Chronic

## Abstract

**Background:**

Fatigue is a highly prevalent and debilitating symptom among patients in the chronic phase of aneurysmal subarachnoid haemorrhage (aSAH) with no identified effective treatment. Cognitive therapy has been shown to have moderate effects on fatigue. Delineating the coping strategies used by patients with post-aSAH fatigue and relating them to fatigue severity and emotional symptoms could be a step towards developing a behavioural therapy for post-aSAH fatigue.

**Methods:**

Ninety-six good outcome patients with chronic post-aSAH fatigue answered the questionnaires Brief COPE, (a questionnaire defining 14 coping strategies and three Coping Styles), the Fatigue Severity Scale (FSS), Mental Fatigue Scale (MFS), Beck Depression Inventory (BDI-II) and Beck Anxiety Inventory (BAI). The Brief COPE scores were compared with fatigue severity and emotional symptoms of the patients.

**Results:**

The prevailing coping strategies were “Acceptance”, “Emotional Support”, “Active Coping” and “Planning”. “Acceptance” was the sole coping strategy that was significantly inversely related to levels of fatigue. Patients with the highest scores for mental fatigue and those with clinically significant emotional symptoms applied significantly more maladaptive avoidant strategies. Females and the youngest patients applied more “Problem-Focused” strategies.

**Conclusion:**

A therapeutic behavioural model aiming at furthering “Acceptance” and reducing passivity and “Avoidant” strategies may contribute to alleviate post-aSAH fatigue in good outcome patients. Given the chronic nature of post-aSAH fatigue, neurosurgeons may encourage patients to accept their new situation so that they can start a process of positive reframing instead of being trapped in a spiral of futile loss of energy and secondary increased emotional burden and frustration.

## Introduction

Fatigue can be defined as a state characterized by a weariness unrelated to previous exertion levels that is usually not ameliorated by rest [12] The literature describes two types of fatigue: physical fatigue and mental fatigue. While physical fatigue relates to muscle performance, mental fatigue is brought on by periods of taxing cognitive processes [19, 38]. Fatigue is a prominent symptom after aSAH with a prevalence of 31–90% in the early phase [26, 50]. Fatigue persists beyond 1 year after the haemorrhage in about one third of the patients and improves only negligible over time [50]. Patients consider post-aSAH fatigue to be among their most disabling symptoms [18, 46, 48]. In contrast to neurological deficits like hemiparesis or aphasia, fatigue is an “invisible” sequel which contributes to variable acknowledgement by family, society and caregivers. Survivors of aSAH reported a lack of awareness regarding the consequences of their haemorrhage and emphasised the importance of multidisciplinary follow-ups, which were found to be mostly missing [38]. Even when post-aSAH fatigue is addressed as a health problem, rehabilitation efforts are limited since there is so far not developed an effective and long-lasting therapeutic intervention for this type of fatigue. Furthermore, those who are seemingly well recovered -good outcome patients with chronic post-aSAH fatigue- receive less support from rehabilitation services and often have only one neurosurgical follow-up consultation. At the same time, this subgroup should have the best resources to subsequently develop strategies to cope with their post-aSAH fatigue.

Coping can be defined as what people do to overcome problems and difficulties, i.e. how they attempt to minimize stress associated with negative experiences. Consciously and subconsciously, individuals use a wide range of coping strategies that may be identified and measured using structured self-report questionnaires [11]. A such commonly used questionnaire, the 28 items Brief-COPE, evaluates how a person uses 14 different coping strategies; strategies that again can be grouped into three overarching Coping Styles; the “Problem-Focused” style, the “Emotion-Focused” style and the “Avoidant” style. Studies in patients with various neurological diseases have shown that the “Problem-Focused” Coping Style is associated with a better health-related quality of life (HRQoL), while the avoidant style has been associated with poorer HRQoL and is considered maladaptive [20, 37, 40]. Results are ambiguous when concerning the association between the “Emotion-Focused” Coping Style and HRQoL [2, 23]. Patients with depression and/or anxiety apply coping more extensively than those without mood symptoms and they tend to use emotional as well as avoidant strategies [1, 20, 37]. Coping Style profiles have been associated with age at ictus [5, 15]. Younger adults tend to apply more problem-oriented strategies, whereas older individuals utilize emotionally oriented strategies [5, 6, 15, 44]. The choice of coping strategies also seems to vary by gender with females applying more emotionally oriented coping [17, 23, 32, 44], this has though not been reproduced in aSAH patients.

Thastum et al. developed a treatment strategy in chronic post-concussion syndrome applying the idea of negative illness perception and maladaptive illness behaviour intended to break the vicious circles of excessive rest due to fatigue by gradually changing attitudes and slowly increasing participation in daily activities [43]. Likewise, cognitive behavioural therapy, graded exercise therapy, and acceptance and commitment therapy has been employed to treat mental fatigue syndrome and fatigue in chronic pain syndromes [7, 8, 21, 38]. Hence, in order to develop similar behavioural therapy approaches customized for post-aSAH fatigue, knowledge of applied coping strategies in that group of patients would be crucial. However, no study has yet assessed coping using the Brief-COPE in aSAH patients, nor has the relationship between coping strategies and post-aSAH fatigue been elucidated. We therefore aim at delineating coping strategies in good outcome patients with post-aSAH fatigue and relate coping to fatigue intensity and emotional symptoms.

## Methods

### Patients

All patients enrolled in a randomized controlled trial (RCT) investigating the effect of a dopaminergic regulator on fatigue were included in the present study [49]. All assessments in the present study were baseline values, collected prior to randomization into one of the treatment arms. The RCT had the ClinicalTrials.gov number NCT03209830 and was approved by the national competent authority and the regional ethic committee (16/-2214). All included patients signed informed consent.

**Fig. 1:**
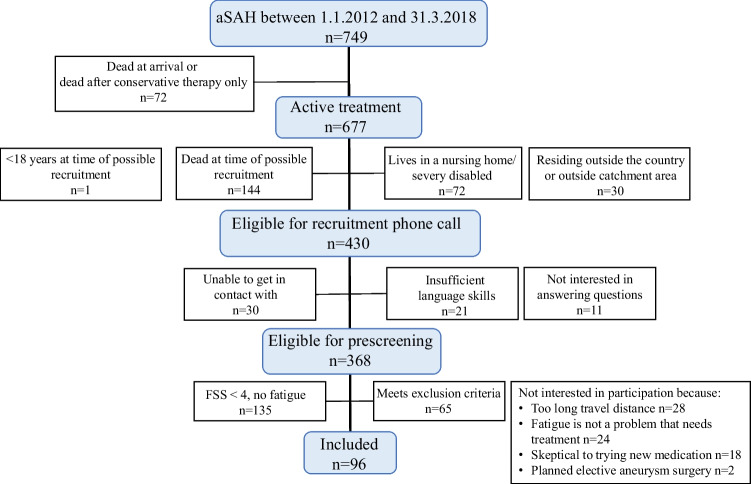
Flowchart of patient inclusion. aSAH: aneurysmal subarachnoid haemorrhage; FSS: fatigue severity scale mean score

For the RCT, patients who had aSAH between January 2012 to March 2018 were screened for inclusion. For recruitment, a neuropsychologist performed telephone interviews and the physician performed the medical screening of eligible candidates for participation. Patients ≥ 18 years at the time of inclusion in the chronic phase of their aSAH (ie. ≥ 12 months since ictus) and with a fatigue severity scale (FSS) mean score ≥ 4 were eligible to participate in the study. Patients were excluded if they had chronic degenerative neurological disease, ongoing epilepsy, or inadequately treated hydrocephalus. Patients were also excluded if they had undergone brain surgery within the past 12 months. Patients who were unable to consent and/or were deemed unable to perform the assessments were excluded. Patients whose language abilities were too poor to understand the assessments were also excluded. Figure 1 shows the flowchart of patient inclusion. Ninety-six patients participated in the study, with 67.7% of the study population being female. Table 1 presents the demography and clinical data of the patients. Median age at the time of inclusion was 58 years (patients ranging from 22 to 74 years), and the median time since inclusion was 26 months (range 12 to 83 months). At 36.5% the rate of return to work was low.

**Table 1:** Included patients

	%
Female/Male	67.7/32.3
Age at inclusion (years)	
< 40	5.2
40–59	55.2
≥ 60	39.6
Age at ictus (years)	
< 40	9.4
40–59	67.7
≥ 60	22.9
Hunt and Hess Score 1/2/3/4/5	26.0/34.4/12.5/16.7/10.4
Modified Fisher Score 1/2/3/4	39.6/5.2/44.8/10.4
Intracerebral hemorrhage	22.9
Aneurysm location	
Anterior circulation	86.5
posterior circulation	13.5
Aneurysm repair surgical/endovascular	44.8/55.2
Cerebrospinal fluid shunt	25
Time from ictus to inclusion (months)	
≤ 24	49.0
25 – 59	33.3
≥ 60	17.7
Work status	
No return to work	63.5
Return to work	36.5
Nicotine status	
Current	40.6
Never	37.5
Former	21.9
Neuropsychological test performance Normal/mild impairment/moderate impairment/deficit (%)	
Sensomotor function	62.7/20.2/8.4/8.7
Executive function	79.2/8.0/5.9/6.9
Attention	78.6/11.6/5.9/3.9
Psychomotor speed	78.4/12.5/3.6/5.5
Verbal learning	56.6/20.1/17.7/5.6
Verbal memory	69.8/13.3/9.4/7.5

Included patients also underwent neuropsychological assessment which confirmed that they were able to answer the questionnaires. We investigated the neuropsychological domains sensorimotor function, executive function, attention, psychomotor speed, verbal learning, and verbal memory. Details about the 24 tests performed in our patients along with the test results have been published previously [48]. Table 1 shows the fractions of normal test performance, mild or moderate impairments, and deficits.

In order to assess their fatigue, coping strategies and emotional symptoms, patients filled out questionnaires in Viedoc™ (Viedoc Technologies ®), which is a widely used, web-based electronic data capture system for managing Case Report Form data in clinical studies and patient registries [47]. A neuropsychologist explained the questionnaires for the patients and they filled them out in their own pace at home. All questions needed to be addressed in order to send the questionnaires and no double marking or commentaries were possible when filling out the questionnaires.

The modified Ranking Score (mRS) [41] was scored by the attending neurosurgeon in a face-to-face outpatient clinic non-structured interview.

## Measurements

- Brief-COPE is a shortened version of the COPE questionnaire, measuring several distinct coping strategies. The questionnaire utilizes a 4-point scale with 1 representing “I haven’t been doing this at all”, 2 “A little bit”, 3 “A medium amount” and 4 representing “I’ve been doing this a lot”. The participant checks off on the score most appropriate for the given statement. Brief COPE consists of 28 statements which are grouped into 14 coping strategies [9] which can be assigned into three Coping Styles [13]. The “Problem-Focused” Coping Style consists of the strategies “Active Coping”, “Informational Support”, “Positive Reframing”, and “Planning”, whereas the “Emotion-Focused” Coping Style comprises the strategies “Emotional Support”, “Venting”, “Humor”, “Acceptance”, “Religion”, and “Self Blame”. The “Avoidant” Coping Style embodies the strategies “Self Distraction”, “Denial”, “Substance Use” and “Behavioural Disengagement”. The score for a coping style is the mean value of the coping strategy scores within each style.
- The Fatigue Severity Scale (FSS) is a questionnaire measuring different facets of fatigue, some of which are fatiguability, impact on physical functioning, frequency and severity. It measures the impact of fatigue on daily living. The questionnaire contains 9 statements [25], which are ranged from 1 to 7 where a higher score indicates larger agreement with the given statement. The FSS score is the mean of the nine statement scores. Only patients with a FSS mean score ≥ 4 were included in the study as this was considered a relevant cut-off [24, 25]. We split the patient group into 2 subgroups with the cut-off set at 6.11, which corresponded to the median FSS score for the patient cohort.
- The Mental Fatigue Scale (MFS) contains 15 statements addressing the mental aspects of fatigue, also covering cognitive and sensory symptoms. These symptoms are also described by their intensity, frequency and duration. Each statement has a value ranging from 0 to 3, where 0 indicates normal function, 1 indicates a problem, 2 indicates pronounced symptoms and 3 indicates maximal symptoms [22]. If two neighbouring statements cover the patient’s symptoms, it is possible to score in between them, resulting in scores of 0.5, 1.5, or 2.5. The scores from statements 1 – 14 are added up and a score ≥ 10.5 is indicative of mental fatigue. Statement 15 asks about diurnal variations and has no numerical value. We split the patient group into 2 subgroups with the cut-off set at ≥ 10.5; i.e. into those with mental fatigue and those without (< 10.5).
- Beck Depression Inventory 2^nd^ edition (BDI-II) is an inventory containing 21 items which assess different symptoms or attitudes associated with depression. These symptoms include different aspects of self-image, impact on daily functioning, and anhedonia among others [4]. Each category is accompanied by 4 or 5 self-evaluative statements whose rank reflects the symptom’s severity. Each statement has a score range between 0 to 3, with higher scores indicating increased severity of the given symptom. The scores are summed, ranging from 0 to 63. The cut-off for clinically significant depression is set at ≥ 20.0 (moderate to severe depressive symptoms).
- Beck’s Anxiety Inventory (BAI) is an inventory measuring different aspects of clinical anxiety, such as catastrophizing, inability to relax, and impact on distinct physiological processes (breathing, digestion, shaking and so on). The inventory contains 21 items that each describes a given symptom [3]. The respondent rates the severity of their symptom using a 4-point scale ranged 0 to 3, 0 indicating ‘not at all’ and 3 indicating ‘I could barely stand it’. The scores are then summed, ranging from 0 to 63. The cut-off for clinically significant anxiety is set at ≥ 16.0 (moderate to severe anxiety symptoms).

### Statistical analysis

Statistical analysis was performed using IBM SPSS Statistics 27 (Armonk, NY: IBM Corp). Continuous variables were presented with median and interquartile range (IQR), while categorical variables were presented with percentage. Groups were compared using Kruskal–Wallis tests and Mann–Whitney U test.

The analysis of coping strategies was conducted by comparing the specific coping strategy scores against the FSS and MFS scores for the whole group (n = 96). Then, coping strategy scores were organized by gender, age at ictus, severity of emotional symptoms, time passed since the ictus and nicotine use. Backward multiple regression was used to identify a parsimonious combination of coping strategies in predicting the FSS mean score and the MFS sum score. Assumptions of linearity, normally distributed errors, and uncorrelated errors were checked and fulfilled for all variables added into the model. Collinearity was checked by help of the tolerance factor which had to exceed 1-r^2^ in order to be accepted as a significant variable. Results were considered significant with two-sided p < 0.05.

## Results

### The prevailing coping strategies

**Fig. 2:**
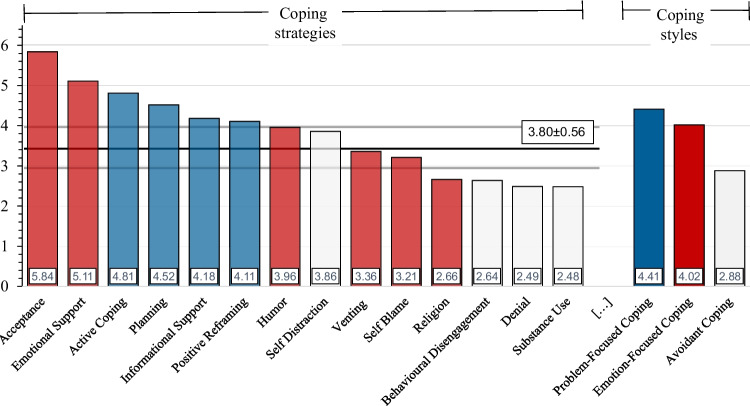
Mean Brief-COPE strategy scores against the mean Brief-COPE score of all statements ± 2SEM. Right 3 columns: Coping Styles. Colour codes: Blue: “Problem-Focused” Coping, Red: “Emotion-Focused” Coping, White: “Avoidant” Coping

Figure 2 shows the scores for the 14 coping strategies and the three coping styles arranged in accordance to their scores. The total mean of all coping strategies was calculated to be 3.80 with a standard error of 0.28. Four coping strategies had scores > the total mean + 2SEM: “Acceptance” (5.84 ± 1.7), “Emotional Support” (5.11 ± 1.76), “Active Coping” (4.81 ± 1.52), and “Planning” (4.52 ± 1,52). The coping strategies with the lowest scores were all “Avoidant” strategies – “Substance Use” (2.48 ± 0.97), “Denial” (2.49 ± 0.77) and “Behavioural Disengagement” (2.62 ± 0.98).

Table 2 shows the scores for the coping strategies and styles along with the scores from the questionnaires for all patients and stratified according to the mRS. There were no differences in relation to mRS.

**Table 2:** Mean and standard deviation or median and interquartile range for the questionnaires in all patients and stratified according to the modified Ranking score (mRS) [41]

	All patients n = 96	mRS 0 n = 5	mRS 1 n = 68	mRS 2 n = 23	p =
Fatigue Severity Scale mean score [25]	5.99 ± 0.79	6.02 ± 0.83	6.02 ± 0.75	5.90 ± 0.91	0.928
Mental Fatigue Scale Sum Score [22]	18.14 ± 5.58	17.30 ± 7.68	17.57 ± 4.88	19.98 ± 6.83	0.327
Beck Depression Inventory 2^nd^ ed. (BDI-II) [4]	15.0 10.0;21.0	15.0 8.0;19.0	14.0 9.25;22.0	17.0 10.0;20.0	0.684
Beck Anxiety Inventory (BAI) [3]	7.0 3.0;13.0	9.0 2.0;11.0	7.0 3.0;13.0	10 6.0;14.0	0.391
Coping strategies					
Accept	5.84 ± 1.70	6.20 ± 1.64	5.66 ± 1.77	6.30 ± 1.46	0.316
Emotional Support	5.11 ± 1.76	5.80 ± 2.28	5.19 ± 1.86	4.74 ± 1.32	0.474
Active Coping	4.81 ± 1.52	4.20 ± 1.64	4.91 ± 1.52	4.65 ± 1.53	0.675
Planning	4.52 ± 1.52	3.60 ± 1.52	4.65 ± 1.54	4.35 ± 1.40	0.312
Informational Support	4.18 ± 1.49	3.40 ± 1.67	4.22 ± 1.53	4.22 ± 1.31	0.444
Positive Reframing	4.11 ± 1.67	3.60 ± 1.52	4.16 ± 1.72	4.09 ± 1.56	0.777
Humor	3.96 ± 1.67	5.00 ± 0.71	3.93 ± 1.78	3.83 ± 1.40	0.155
Self Distraction	3.86 ± 1.41	3.40 ± 1.34	3.84 ± 1.41	4.04 ± 1.46	0.659
Venting	3.36 ± 1.20	3.60 ± 1.14	3.32 ± 1.28	3.43 ± 0.99	0.620
Self Blame	3.21 ± 1.47	2.80 ± 1.10	3.22 ± 1.52	3.26 ± 1.46	0.838
Religion	2.66 ± 1.07	2.80 ± 1.10	2.68 ± 1.10	2.57 ± 0.99	0.755
Behavioural Disengagement	2.64 ± 0.98	2.50 ± 0.58	2.53 ± 0.89	3.00 ± 1.21	0.220
Denial	2.49 ± 0.77	2.80 ± 1.10	2.50 ± 0.76	2.39 ± 0.73	0.633
Substance use	2.48 ± 0.97	2.20 ± 0.45	2.37 ± 0.85	2.87 ± 1.29	0.102
Coping Styles					
Problem-Focused Coping	4.41 ± 1.25	3.70 ± 1.20	4.48 ± 1.29	4.33 ± 1.13	0.328
Emotion-Focused Coping	4.02 ± 0.81	4.37 ± 0.96	4.00 ± 0.83	4.02 ± 0.74	0.618
Avoidant Coping	2.88 ± 0.71	2.88 ± 0.66	2.81 ± 0.69	3.08 ± 0.78	0.339

### Gender based differences in the use of coping strategies

**Fig. 3:**
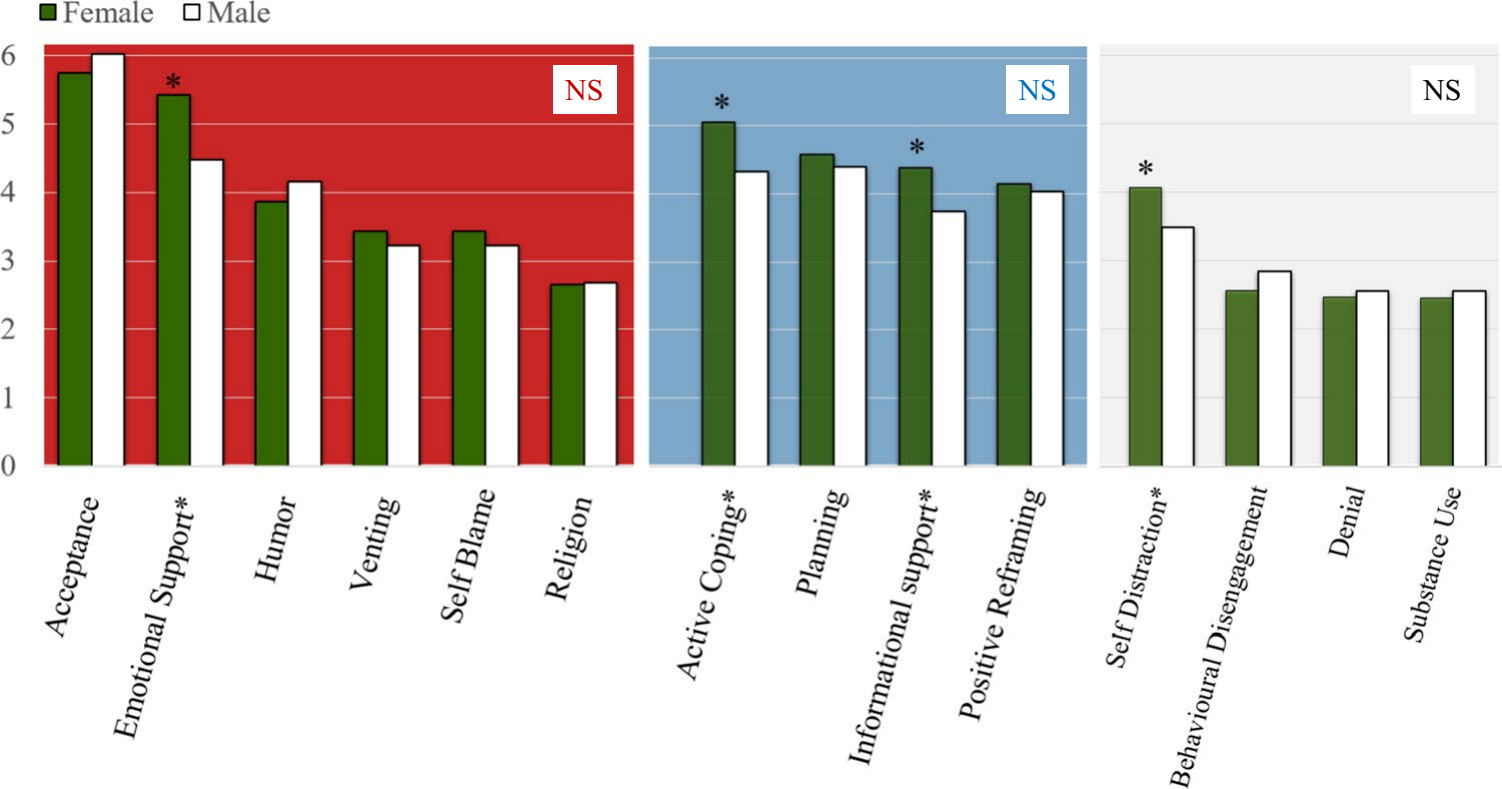
Mean Brief-COPE item scores stratified by gender. Background colours indicate coping strategies within the coping styles “Emotion-Focused” Coping (red), “Problem-Focused” Coping (blue), and,“Avoidant” Coping (grey). *: p < 0.05; NS: not significant

There were gender differences in the application of coping strategies (Fig. 3). Females scored significantly higher than males in 4 strategies: “Emotional Support” (5.42 (F) vs 4.48 (M), p = 0.014), “Active Coping” (5.05 (F) vs 4.32 (M), p = 0.019), “Informational Support” (4.34 (F) vs 3.74 (M), p = 0.005) and “Self Distraction” (4.06 (F) vs 3.48 (M), p = 0.044) (Fig. 3).

There were no gender-based differences when looking at the three Coping Styles. There was no significant difference in fatigue between females and males (FSS mean score: 5.94 ± 0.82 (F) vs 6.10 ± 0.71 (M), p = 0.347; MFS sum score: 18.46 ± 5.52 (F) vs 17.45 ± 5.74, p = 0.418).

### Use of coping strategies by age at ictus

Coping strategies were applied differently depending on age at ictus. Scores were significantly higher in the youngest patients for the coping strategies “Active Coping”, “Positive Reframing”, “Venting”, and “Denial” (Table 3). There were no age differences in scores for the two most prevailing coping strategies “Acceptance” and “Emotional Support”.

**Table 3:** Mean and standard deviation categorized by age at ictus

	< 40 years	40–59 years	≥ 60 years	p =
Fatigue Severity Scale mean score	6.11 ± 0.85	5.95 ± 0.83	6.06 ± 0.64	0.795
Mental Fatigue Scale sum score	19.78 ± 5.20	17.95 ± 5.29	18.02 ± 6.64	0.429
Coping Strategies				
Acceptance	5.67 ± 2.0	5.88 ± 1.67	5.82 ± 1.74	0.979
Emotional Support	4.78 ± 1.92	5.22 ± 1.82	4.95 ± 1.59	0.733
Active Coping	5.22 ± 1.20	5.03 ± 1.46	4.00 ± 1.60	0.016
Planning	4.89 ± 1.17	4.65 ± 1.52	4.00 ± 1.57	0.191
Informational Support	4.78 ± 1.48	4.25 ± 1.47	3.73 ± 1.49	0.180
Positive Reframing	5.56 ± 2.13	4.08 ± 1.51	3.64 ± 1.65	0.034
Humor	4.89 ± 2.21	3.98 ± 1.6	3.50 ± 1.54	0.166
Self Distraction	4.78 ± 1.48	3.88 ± 1.4	3.45 ± 1.3	0.064
Venting	4.33 ± 1.66	3.37 ± 1.11	2.95 ± 1.05	0.029
Self Blame	4.00 ± 2.18	3.23 ± 1.47	2.82 ± 1.01	0.405
Religion	2.78 ± 1.09	2.60 ± 1.09	2.77 ± 1.02	0.592
Behavioural Disengagement	2.44 ± 0.88	2.75 ± 0.99	2.38 ± 0.97	0.103
Denial	2.67 ± 0.71	2.58 ± 0.83	2.14 ± 0.47	0.023
Substance Use	2.33 ± 0.71	2.52 ± 1.00	2.41 ± 1.01	0.809
Coping Styles				
Problem Focused Coping	5.11 ± 1.27	4.50 ± 1.18	3.84 ± 1.27	0.026
Emotion Focused Coping	4.41 ± 1.08	4.05 ± 0.75	3.80 ± 0.82	0.261
Avoidant Coping	3.06 ± 0.56	2.93 ± 0.75	2.62 ± 0.63	0.101

The “Problem-Focused” Coping Style decreased with age (p = 0.026) (Table 3).

### Coping strategies and coping styles versus median FSS score

Except for the coping strategies “Acceptance” (p = 0.048) and “Substance use” (p = 0.008), there were no significant differences between coping strategies in those with FSS mean score < 6.11 versus those with the most pronounced fatigue as expressed with a FSS mean score ≥ 6.11. Backward multiple regression revealed a model with the most parsimonious variables “Venting”, “Self Distraction”, “Substance use”, and “Acceptance” F(4,90) = 3.996, p = 0.005, adjusted r^2^ = 0.113. The equation for the model was: FFS mean score = 5.927 + 0.228 “Venting” + 0.280 “Substance use” – 0.191 “Self Distraction”—0.215 “Acceptance” + *e*. “Acceptance” (F = -2.182, p = 0.032) was though the only significant contributor in the model.

Fatigue severity as measured by FSS mean score had no significant relation to any of the 3 Coping Styles.

### Coping strategies and coping styles in MFS sum score < 10.5 versus MFS sum score ≥ 10.5

**Fig. 4:**
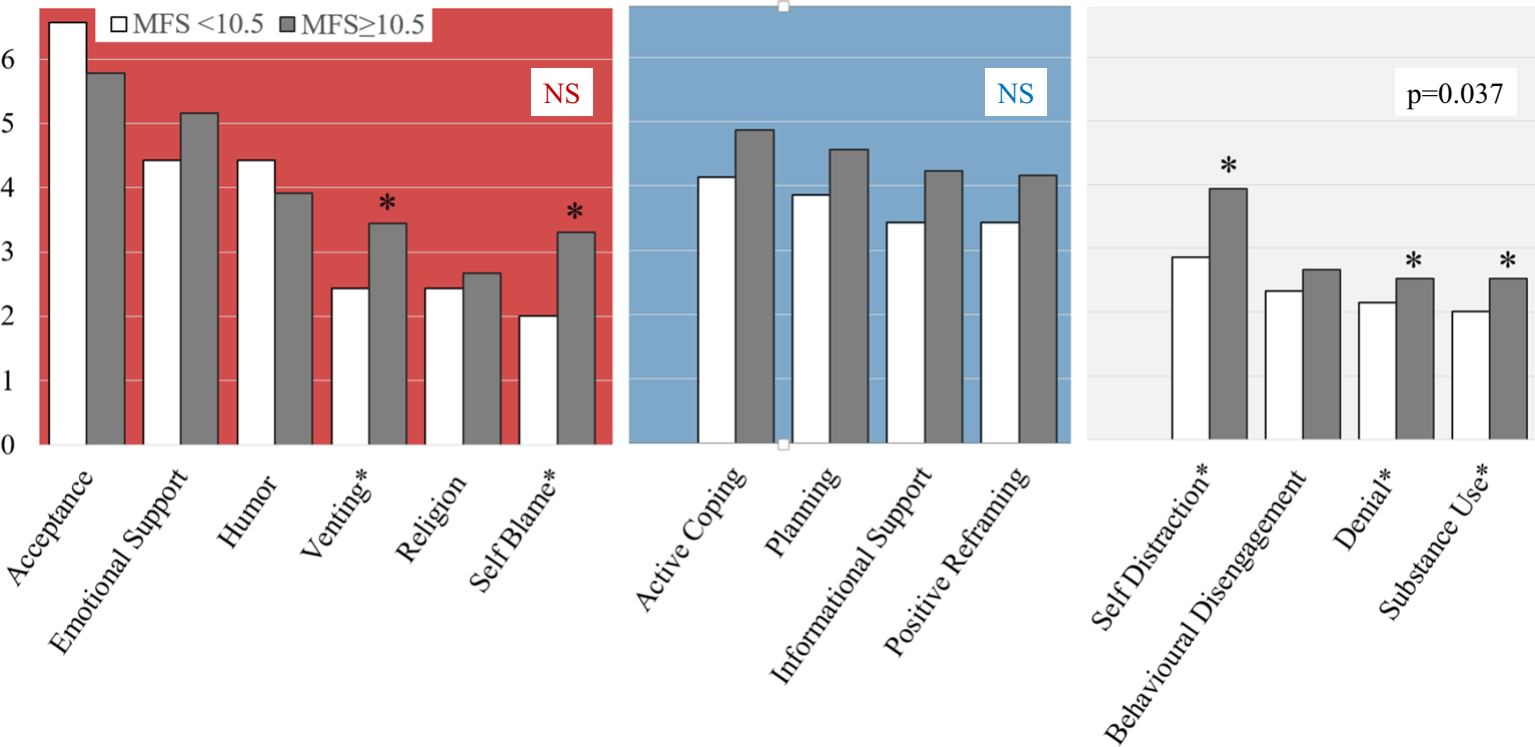
Mean Brief-COPE item scores when stratifying the study population by Mental Fatigue Scale (MFS) sum scores indicating no mental fatigue (MFS < 10.5) and significant mental fatigue (MFS ≥ 10.5). Background colours indicate coping strategies within the coping styles “Emotion-Focused” Coping (red), “Problem-Focused” Coping (blue), and, “Avoidant” Coping (grey). *: p < 0.05; NS: not significant

The patients with clinically significant fatigue (MFS sum score ≥ 10.5) scored higher for the “Avoidant” strategies “Self Distraction” (p = 0.009), “Denial” (p = 0.023), and “Substance Use” (p < 0.001), as well as for the emotional-focused strategies “Venting” (p = 0.007) and “Self Blame” (p = 0.012) (Fig. 4). Consequently, this subgroup scored significantly higher for the “Avoidant” Coping Style (p = 0.037). Those who did not score positive for mental fatigue (MFS sum score < 10.5) had higher scores for “Acceptance”, the difference, however, was only borderline significant (p = 0.064).

Backward multiple regression revealed a model with the most parsimonious variables “Emotional Support”, “Venting”, “Substance Use” and “Acceptance”, F(4,90) = 8.581, p < 0.001, adjusted r^2^ = 0.234. The equation for the model was MFS sum score = 11.165 + 0.217 “Emotional Support” + 0.260 “Venting” + 0.294 “Substance use” – 0.245 “Acceptance” + *e*. All variables in the final model were significant (“Emotional Support” t = 2.154, p = 0.034; “Venting” t = 2.700 p = 0.008; “Substance Use” t = 3.185 p = 0.002; “Acceptance” t = -2.528 p = 0.002).

When entering coping styles into backward multiple regression we obtained a model with F(1,93) = 16.462, p < 0.001, with avoidant coping style being the only significant variable (t = 4.057, p < 0.001).

### Emotional symptoms and use of coping strategies

Clinical depression (BDI-II score ≥ 20) was present in 27 (28.1%) patients, whereas 15 (15.6%) patients had clinical anxiety (BAI score ≥ 16), see also Table 4.

**Table 4:** Mean and standard deviation for the Beck Anxiety Inventory (BAI) and the Beck Depression Inventory 2^nd^ Ed. (BDI-II) stratified by clinically significant anxiety (BAI ≥ 16) and depression (BDI-II ≥ 20) [3, 4]

	BAI < 16 N = 81	BAI ≥ 16 N = 15	p =	BDI-II < 20 N = 69	BDI-II ≥ 20 N = 27	p =
Fatigue Severity Scale mean score	5.93 ± 0.8	6.30 ± 0.66	0.099	5.81 ± 0.79	6.46 ± 0.55	< 0.001
Mental Fatigue Scale sum score	17.40 ± 5.47	22.10 ± 4.50	0.001	16.59 ± 5.35	22.09 ± 4.05	< 0.001
Coping Strategies						
Acceptance	5.91 ± 1.7	5.47 ± 1.7	0.329	6.16 ± 1.63	5.04 ± 1.63	0.002
Emotional Support	5.15 ± 1.81	4.93 ± 1.81	0.722	5.23 ± 1.74	4.81 ± 1.82	0.351
Active Coping	4.74 ± 1.53	5.20 ± 1.53	0.353	4.62 ± 1.52	5.30 ± 1.46	0.054
Planning	4.37 ± 1.52	5.33 ± 1.52	0.031	4.28 ± 1.50	5.15 ± 1.38	0.015
Informational Support	4.15 ± 1.54	4.33 ± 1.54	0.682	4.07 ± 1.43	4.44 ± 1.63	0.372
Positive Reframing	4.04 ± 1.71	4.53 ± 1.71	0.177	3.91 ± 1.63	4.63 ± 1.67	0.047
Humor	3.98 ± 1.64	3.87 ± 1.64	0.630	3.84 ± 1.58	4.26 ± 1.87	0.425
Self Distraction	3.64 ± 1.32	5.07 ± 1.32	< 0.001	3.49 ± 1.30	4.81 ± 1.24	< 0.001
Venting	3.20 ± 1.08	4.27 ± 1.08	0.002	3.00 ± 0.86	4.30 ± 1.44	< 0.001
Self Blame	2.95 ± 1.31	4.60 ± 1.31	< 0.001	2.75 ± 1.18	4.37 ± 1.52	< 0.001
Religion	2.54 ± 1.04	3.27 ± 1.04	0.002	2.49 ± 1.05	3.07 ± 1	< 0.001
Behavioural Disengagement	2.61 ± 0.93	2.80 ± 0.93	0.673	2.49 ± 0.84	3.04 ± 1.19	0.023
Denial	2.43 ± 0.72	2.80 ± 0.72	0.087	2.32 ± 0.58	2.93 ± 1	0.003
Substance Use	2.31 ± 0.70	3.40 ± 0.70	< 0.001	2.25 ± 0.63	3.07 ± 1.39	< 0.001
Coping Styles						
Problem-Focused	4.32 ± 1.29	4.85 ± 1.25	0.122	4.22 ± 1.25	4.88 ± 1.15	0.016
Emotion-Focused	3.95 ± 0.83	4.40 ± 0.81	0.044	3.91 ± 0.76	4.31 ± 0.88	0.027
Avoidant Coping	2.76 ± 0.63	3.52 ± 0.71	< 0.001	2.64 ± 0.56	3.46 ± 0.74	< 0.001

As shown in Table 4, there were significant differences between the groups without and with moderate to severe depressive symptoms (BDI-II score ≥ 20) for seven different coping strategies. While patients without clinical depression scored significantly higher for “Acceptance” (p = 0.00), they scored significantly lower for the strategies “Venting” (p = 0.001), “Self Distraction” (p = 0.001), “Self Blame” (p = 0.000), “Behavioural Disengagement” (p = 0.002), “Denial” (p = 0.000) and “Substance Use” (p = 0.001) than those with moderate to severe depressive symptoms. Those with clinical depression hence scored significantly higher on the “Avoidant” Coping Style as compared to those without clinical depression (p = 0.000).

Patients with moderate to severe symptoms of anxiety (BAI score ≥ 16) scored significantly higher for six coping strategies: “Self Distraction” (p = 0.000), “Venting” (p = 0.000), “Self Blame” (p = 0.000), “Substance Use” (p = 0.001), “Planning” (0.018) and “Religion” (p = 0.01), see Table 4.

Patients with moderate to severe symptoms of anxiety scored significantly higher than those without or mild anxiety for the “Avoidant” Coping Style (p = 0.001) and the “Emotion-Focused” Coping Style (p = 0.038).

### Differences in coping strategies based on time passed since the ictus, nicotine use and work status

Time after ictus had little impact on coping strategies, with only the use of “Venting” increasing over time (mean score in recent phase 2.25, in moderate phase 3.36 and in chronic phase 3.65, p = 0.048). Likewise, no clear patterns were found for coping strategies or Coping Styles in relation to work status or smoking habits/nicotine use.

## Discussion

The aim of the present study was to delineate coping strategies in good outcome patients with post-aSAH fatigue and relate coping to fatigue intensity and emotional symptoms.

We found that “Acceptance” was the prevailing coping strategy in that subgroup of aSAH survivors and that they applied mostly “Problem-Focused” strategies. “Acceptance” was significantly inversely related to levels of fatigue. Patients with the highest scores for mental fatigue and those with significant emotional symptoms applied maladaptive strategies significantly more. Our females with post-aSAH fatigue applied more “Problem-Focused” coping strategies which opposes the gender theories positing that women apply mostly “Emotion-Focused” strategies when dealing with stressors [20]. Gender based coping after aSAH has, however, merely been investigated in one smaller study which failed to find differences [44]. Our findings also indicate that younger persons applied more “Problem-Focused” strategies than older subjects. This corroborates the findings of Tomberg et al. who compared aSAH patients younger than 50 years versus older ones [44]. The pattern of the applied coping strategies was not influenced by time passed since the ictus, which corroborates with the chronic nature of both fatigue and emotional symptoms persisting quite unchanged over many years after the aSAH [42, 48].

### Coping after aSAH

Studies on coping after aSAH are relatively scarce. A qualitative study investigating patients six years after their aSAH identified receiving support from family, society, employers, or technical equipment to be a core coping strategy [38]. Tomberg et al. [44] found that patients after SAH applied social support strategies less than normal controls with a tendency of using acceptance oriented strategies. In the present study we do not know to which extent our patients had received support and we cannot rule out that different levels of supportive backup could have an impact on choice of coping strategies and levels of fatigue. Likewise, socioeconomic factors and personality traits may play a role in coping patterns after aSAH. This has not been studied, but patients with higher levels of education generally tend to have better outcomes [18]. Noble et al. described the cluster of sequels after aSAH in good outcome patients as post-traumatic stress syndrome and found that its development was promoted by maladaptive coping strategies [33]. ASAH patients seem to use adaptive coping strategies like “Acceptance”, “Emotional Support”, “Active Coping” and “Planning” which also have been found to transcend other neurological disorders [23, 31, 35].

This pattern of preferred coping strategies also seems to mirror that of the general population as it concurs with the findings in a study examining the use of coping strategies in a large Chilean sample (n = 1847) [16]. Furthermore, “Acceptance”, “Emotional Support”, “Active Coping” and “Planning” emerged as the most important coping strategies used by medical students when handling their stress [32]. Even though coping strategies are used similarly, the extent to which they are applied seems to be more extensive in aSAH patients than in normal controls [20] or in a healthy population that has been exposed to a stressful event [16]. Hedlund et al. [20] also found that those using coping most extensively had the poorest HRQoL. Considering that “Acceptance” is one of the most applied strategies in general, it is not a paradox that we find high scores for this strategy and at the same time a significant inverse relation to levels of fatigue. This relation to fatigue was unique among the 14 strategies investigated and designates “Acceptance” as a target for therapeutical measures.

### Coping Strategies and Fatigue

The coping strategy «Acceptance» has been found to improve HRQoL through emotional, behavioural, and cognitive processes [10, 30]. “Acceptance” associates with better psychological and physical health in diseases which course is perceived as uncontrollable, such as chronic fatigue syndrome and chronic pain [7, 30, 45]. This notion concurs with our finding that “Acceptance” scores were inversely related to both the FSS mean score and the MFS sum score. Consequently, furthering “Acceptance” as a coping strategy may also prove beneficial in patients suffering from post-aSAH fatigue, even though a direct causality cannot be derived from our findings and the association we saw may be caused by a common background factor not controlled for.

Patients with the highest MFS sum scores applied more of the avoidant strategies “Denial” and “Self Distraction”. Denial can exacerbate the impact of the stressor and contribute to psychological distress [9, 26], so that the denial of fatigue may increase its toll. One may argue that “Self Distraction” is a form of denial with similar deleterious effects leading to decreased psychological well-being and poorer psychosomatic health [27, 51]. “Self Distraction” can divert the patient’s cognitive and emotional resources from coping with their problem.

### Interrelation between Coping Strategies, Emotional Symptoms and Fatigue

The rate of depression and anxiety in the present study was in line with earlier reports on mood disturbances in post-aSAH patients [37, 42]. A recent review covering more than 6000 aSAH patients reported that the frequency of depression ranges from 0% to 61.7%, with a weighted proportion of 28.1% [42], which is exactly the frequency of depression in our cohort. The subgroup of aSAH patients with mood disturbances apply coping more extensively, and preferably use passive and avoidant strategies which was found to lead to poorer HRQoL [1, 20, 37]. This is in consensus with our finding of those with mood disorders applying more avoidant strategies. This subgroup also scored significantly higher on the two emotional strategies “Venting” and “Self Blame”. Both are considered maladaptive strategies [27, 51] – a form of rumination – as they focus extensively on negative emotions in a non-productive way, thus aggravating negative feelings [34].

One may note the overlap of increased use of maladaptive strategies in our patients with emotional problems and those with severe mental fatigue. Previous studies have found that the severity of fatigue is increased in patients with emotional symptoms [36, 49]. While fatigue may exist independently of both depression and anxiety, it is likely that they can be caused or maintained by some of the same cognitive processes [48] and hence be both modifyable by targeted behavioural therapy.

### Implications

Acceptance is a multidimensional construct and should not be apprehended as “giving-up” but rather as an active process of re-evaluation of possibilities and life priorities [45]. Acceptance acknowledges the new reality and helps to discard efforts that are not working so that workable efforts can be pursued and meaningful goals achieved [28]. Too actively fighting an uncontrollable symptom can worsen fatigue [28]. Fatigue is perceived as uncontrollable [45]. Attempts to control the uncontrollable may fuel frustration, distress, and hypervigilance to symptoms [45]. Acceptance has been found to be linked to low levels of concurrent distress and was found to be a prospective predictor of low distress in cancer patients [10]. Furthermore, acceptance was associated with lower levels of fatigue, functional impairment, and psychological distress in patients with CFS [45].. From this they concluded that “promoting acceptance in patients with CFS may be more beneficial than trying to control largely uncontrollable symptoms” [45]. There are a number of acceptance-based therapies, including cognitive behavioural therapy (CBT), acceptance and commitment therapy, and mindfulness-based stress reduction [28] that have been applied in CFS and fatigue in chronic pain.

Brooks et al. reported that lack of acceptance was associated with the level of fatigue in patients suffering from CFS [7]. After treatment with CBT, their patients showed significantly increased acceptance, less fatigue, reduced impairment of physical functioning, as well as improved work and social adjustment [7]. If such therapies are suitable for the post-aSAH population, however, remains to be investigated. Furthermore, access to this type of therapeutic interventions may be limited. In particular, good outcome aSAH patients often are not enrolled into rehabilitation programs which may include or refer to CBT treatment. The good outcome subgroup often only sees their neurosurgeon in a single outpatient consultation 3–6 months after the ictus and their general practitioner. The latter usually will meet 0 to 2 post aSAH patients through their career and will hence not know what to expect regarding functional outcome, far less know how to guide the patients [39]. Information about the frequency and chronic nature of fatigue can be provided by the neurosurgeon.

The first step to promote acceptance is adequate information and to convey realistic expectations. It is counterproductive to advise patients that they will get much better simply by waiting long enough as it is an avoidant, maladaptive strategy that hinders more proactive mechanisms to deal with the new situation. Patients trust neurosurgeons as the most competent in the matter of their disease and when a promised spontaneous resolve or improvement does not happen, they may become afraid and distrustful, which may aggravate their situation. Information provision after aSAH has been found to be poor and not presented in a patient-friendly manner [14].

Apart from information by the neurosurgeon, departments may develop information booklets that are routinely handed out to patients and their next of kin. Information on a website and an open access to advice and support have also been established in some centres to satisfy the unmet needs of aSAH survivors [14, 38]. Such strategies signal acknowledgement of fatigue as a serious sequel after aSAH, which per se should help patients to accept and cope, thereby alleviating possible secondary distress produced by not being believed by the surroundings.

### Limitations

There are several limitations to our study. Based on the strict inclusion criteria, our patient group was highly selected and the generalizability of our findings is thereby limited. A higher number of participants could possibly have disclosed more significant interrelations between fatigue severity and coping strategies. This is in particular the case for the subgroup analysis like gender- or age-based group differences. Including patients without fatigue after aSAH would have added valuable information as those without chronic fatigue may utilise different coping strategies. Our findings in those with MFS Sum score < 10.5 may indicate so. All our patients were good outcome and independent in activities of daily living. Dependent aSAH survivors were found to apply strategies within problem-oriented coping style significantly less than their independent counterparts [44].

Fatigue is complex, subjective, and difficult to quantify. There is no instrument to measure and fully cover all facets of this entity. We used the FSS because it is one of the most widely used questionnaires that is best validated. It has been found to have excellent internal consistency and test–retest reliability in normal controls as well as in individuals with post-stroke fatigue [29]. The MFS covers more dimensions of fatigue with a stronger focus on mental fatigue which prevails in aSAH patients [48]. Its psychometric properties, however, are not yet extensively studied in the aSAH population. The MFS also incorporates affective, cognitive and sensory symptoms which may be directly linked to fatigue, but could also be distinctive constructs [22]. Theoretically, the MFS sum could hence be higher in the presence of symptoms other than fatigue per se. When relating MFS sum scores to coping strategies this may provide a wider perspective of post-aSAH sequels.

All our patients had volunteered for a RCT, i.e. they can be assumed to be proactive and seeking a solution for their fatigue problem. Our cohort may hence differ from the general post-aSAH fatigue group in terms of coping strategies. On the other hand, we included patients over a wide spectre of age, time passed since the ictus, of typical gender distribution from the entire spectre of aSAH severity, and with typical frequency of emotional symptoms. Furthermore, answering the questionnaires electronically provided a high data quality without missing points.

To develop a complete model of CBT one should also adjust for potential confounders such as recent life events, education level, personality, and level of social and economic support. Future studies could opt at including these variables. They should also include dependent aSAH survivors and good outcome patients without fatigue. Nevertheless, the present study reflects coping strategies and their relation to levels of fatigue in the subgroup of good outcome patients with chronic post-aSAH fatigue, a subgroup that usually has less access to rehabilitation programs. They can be assumed to have considerable unmet needs and could profit from the knowledge gained in our study.

## Conclusion

A therapeutic behavioural model aiming at furthering “Acceptance” and reducing “Avoidant” strategies may contribute to alleviate post-aSAH fatigue in good outcome patients. Given the chronic nature of post-aSAH fatigue, neurosurgeons may encourage their patients to accept their new situation so that they can start a process of positive reframing instead of becoming trapped in a spiral of further futile loss of energy and secondary increased emotional burden and frustration.

## Data Availability

The datasets generated during and/or analysed during the current study are available from the corresponding author on reasonable request.

## Abbreviations

aSAH: Aneurysmal Subarachnoid Haemorrhage
BAI: Beck Anxiety Inventory
BDI-II: Beck Depression Inventory, 2^nd^ edition
CBT: Cognitive behavioural therapy
CFS: Chronic fatigue syndrome
FSS: Fatigue Severity Scale
HRQoL: Health related quality of life
MFS: Mental Fatigue Scale
mRS: Modified Rankin Score
RCT: Randomised Controlled Trial
TBI: Traumatic Brain Injury
